# Beneficial Effects of Soybean-Derived Bioactive Peptides

**DOI:** 10.3390/ijms22168570

**Published:** 2021-08-09

**Authors:** Il-Sup Kim, Woong-Suk Yang, Cheorl-Ho Kim

**Affiliations:** 1 Advanced Bioresource Research Center, Kyungpook National University, Daegu 41566, Korea; 92kis@hanmail.net; 2 Nodaji Co., Ltd., Pohang 37927, Gyeongsangbuk-Do, Korea; 3 Molecular and Cellular Glycobiology Unit, Department of Biological Sciences, SungKyunKwan University, Seoul 16419, Gyunggi-Do, Korea; 4 Samsung Advanced Institute of Health Science and Technology, Seoul 16419, Gyunggi-Do, Korea

**Keywords:** soybean, bioactive peptide, positive effect, human health

## Abstract

Peptides present in foods are involved in nutritional functions by supplying amino acids; sensory functions related to taste or solubility, emulsification, etc.; and bioregulatory functions in various physiological activities. In particular, peptides have a wide range of physiological functions, including as anticancer agents and in lowering blood pressure and serum cholesterol levels, enhancing immunity, and promoting calcium absorption. Soy protein can be partially hydrolyzed enzymatically to physiologically active soy (or soybean) peptides (SPs), which not only exert physiological functions but also help amino acid absorption in the body and reduce bitterness by hydrolyzing hydrophobic amino acids from the C- or N-terminus of soy proteins. They also possess significant gel-forming, emulsifying, and foaming abilities. SPs are expected to be able to prevent and treat atherosclerosis by inhibiting the reabsorption of bile acids in the digestive system, thereby reducing blood cholesterol, low-density lipoprotein, and fat levels. In addition, soy contains blood pressure-lowering peptides that inhibit angiotensin-I converting enzyme activity and antithrombotic peptides that inhibit platelet aggregation, as well as anticancer, antioxidative, antimicrobial, immunoregulatory, opiate-like, hypocholesterolemic, and antihypertensive activities. In animal models, neuroprotective and cognitive capacity as well as cardiovascular activity have been reported. SPs also inhibit chronic kidney disease and tumor cell growth by regulating the expression of genes associated with apoptosis, inflammation, cell cycle arrest, invasion, and metastasis. Recently, various functions of soybeans, including their physiologically active functions, have been applied to health-oriented foods, functional foods, pharmaceuticals, and cosmetics. This review introduces some current results on the role of bioactive peptides found in soybeans related to health functions.

## 1. Introduction

Soybeans contain lipids (20%), proteins (40%), carbohydrates (35%), and other substances (5%). The addition of 12–14% moisture is appropriate for stability during storage. The other substances consist of vitamins and minerals as minor components of the body. Recently, considerable attention has been given to soybeans as a functional food because they have been reported to contain phytochemical substances that prevent cancer and other chronic diseases [1]. Detailed studies of soybeans have identified at least 14 phytochemicals, such as carotenoids, coumarins, flavonoids, lignans, phytic acid, triterpenes, and phenolics, which are involved in cancer prevention [2]. Furthermore, protease inhibitors, oligosaccharides, and dietary fibers are also known to exhibit similar physiological functions [3]. Soybeans contain phytochemicals that possess anticancer, antiaging, antirenal failure, antiobesity, and anticholesterolemic activities. These phytochemicals can also inhibit HIV; prevent gallstone formation, senile dementia, and hyperlipidemia; promote diuretic action; suppress arteriosclerosis, provide relief from constipation; and prevent cardiovascular disease [4,5,6,7]. Therefore, soybeans are involved in the prevention of chronic diseases. Soybeans also contain substances that contribute to intestinal regulation, exert antioxidative properties, prevent osteoporosis, lower blood pressure, exert antithrombotic effects, boost immunity, and promote liver function [4,8,9].

Soybeans are an excellent food source as they contain dietary fiber, qualified protein, a high level of unsaturated fatty acids, and other substances that exert a variety of physiological effects [7,10,11]. In recent years, the Korean population has adopted a Western diet, and the prevalence of certain diseases caused by obesity, such as diabetes or cardiovascular events, has increased [12]. The overconsumption of animal-based foods is a significant cause of obesity, which highlights the need to consume more plant-derived food products such as soy [13]. Compared with other types of beans, soybeans contain 40% protein, which is significantly high. The soy protein is relatively equivalent to that found in meats, dairy, and eggs, but soybeans do not contain cholesterol or saturated fatty acids [14,15]. In October 1999, the Food and Drug Administration of the USA declared that soy protein reduces the danger associated with metabolic dysfunction-related human diseases such as cardiovascular and chronic kidney disease and granted the use of health labels to increase the consumption of soybeans in public interest in the USA and Japan [16,17]. It has been determined that obesity can be effectively prevented and treated by the administration of soy protein. The protein inhibits fat accumulation, increases fat metabolism, and lose body weight by regulating the expression of genes associated with appetite-repressing factors [18,19]. Furthermore, the fermentation of soybeans using active bacteria, such as *Bacillus* spp., produces certain enzymes and other physiochemical substances, which initially are not present. Soybean fermentation is accomplished by either *B. subtilis* or *B. licheniformis*. This fermentation attentuates the spoiling potentials of gut microbiota and tolerates infection by pathogenic bacteria via the absorption of toxic molecules in humans [5]. This review summarizes the physiological activity of soy protein-based bioactive peptide and determines its potential as a “well-being” food.

## 2. Production of Soy Bioactive Peptide

Functional foods such as soybean-derived peptides (SPs) can prevent and treat certain diseases in addition to providing nutrition. Research on soy products has increased significantly as they have emerged as functional foods that improve blood circulation and intestinal regulation. In addition to their nutritional value, soybeans contain specific phytochemical substances that have been known to promote health, including dietary fiber, isoflavones (genistein and daidzein), phospholipids, phenolic acids, trypsin inhibitor, saponins, and phytic acid [10,11,20]. These substances are effective in preventing chronic diseases, including arteriosclerosis, cardiac diseases, diabetes, senile dementia, cancer, and osteoporosis. Furthermore, soybeans have been reported to attenuate fibrinolytic activity, control blood pressure, improve lipid metabolism, and exert antimutagenic, anticarcinogenic, and antibacterial effects [21,22,23,24,25].

SPs are small protein fragments generated by in vitro enzymatic hydrolysis, fermentation (e.g., lactic acid bacteria-mediated fermentation), food processing (e.g., pH modification, heat treatment, protein isolation, ultra-high-pressure processing, and storage conditions) [26,27], and gastrointestinal digestion (specific and nonspecific proteases from the stomach, small intestine, and pancreas, including pepsin, trypsin, chymotrypsin, and pancreatin) of larger soybean proteins, which are beneficially associated with a multitude of metabolic activities [10,11]. The peptide composition is affected by enzymatic hydrolysis or bacteria-mediated fermentation and is also related to the type of soy protein [10]. SPs with different compositions have also been known to exhibit various functional properties with respect to quality, yield, and texture during tofu production [28]. Bioactive peptides comprising 2–20 amino acids are inactive when they are part of the parental protein sequence but become activated upon release by the methods described above [10,28,29].

## 3. Characteristics of Soybean Peptides

SPs may not seem very familiar, but they are quite common. For example, miso, soy sauce, and natto are examples of foods that contain fermented soybeans. These foods contain SPs in trace amounts [30]. Even if one consumes soy protein-containing foods, such as soy milk or tofu, processed without fermentation, SPs are produced in the body by digestive enzymes [10]. Recently, a study on the functional ingredients of soybeans revealed that many functional beverages and foods have been developed that include SPs along with soy isoflavones, dietary fiber, and soybean oligosaccharides [16].

SPs are substances produced during the process of protein degradation. Proteins in food are broken down into amino acids by digestive enzymes in the body while passing through the digestive tract and are absorbed into the small intestine. However, at this time, all proteins are not completely degraded into amino acids and some are absorbed in the form of peptides in which several amino acids are bound [10]. Bioactive SPs are found in part of the parental protein sequence but inactive in those structures [3]. However, released parts are active by enzyme digestion or fermentation [3]. The bioactive compound to which these amino acids are bound is usually a peptide [31,32]. In other words, it can be said to be an intermediate product of proteins and amino acids, and SPs are peptides produced by enzymatic digestion or fermentation of soybean proteins [22].

SPs are hydrolyzed by combining soybean proteins with enzymes, and the resulting oligopeptides are composed of several amino acids. The physical characteristics of SPs include (1) high-concentration dissolution with low viscosity, (2) high clarity, and (3) low allergy compared with soy protein. In addition, purified SP has faster absorption into the body than amino acids [33,34]. In a recent study, the small intestine was shown to follow a separate path to absorb unmodified peptides, and it was found that relatively low-molecular peptides are absorbed [35].

## 4. Functionality of Soy Peptides

Peptides represent a combination of various amino acid chains that form a polymer with a molecular weight of <10,000 Da [10]. Peptides can be mass-produced with high purity through the development of synthetic methods and are starting to attract attention as important highly functional raw materials due to advances in molecular biology that have allowed the elucidation of in vivo protein synthesis mechanisms [3,10,36,37]. Bioactive peptide drugs have several advantages over low-molecular organic drugs [11]. Peptides are broken down to individual amino acids, reducing the risk of toxicity, and because they have a short half-life, they do not accumulate in tissues [3]. Peptides can be dissolved at high concentrations in a low-viscosity state and can maintain high clarity (clear solution) [14,36,37]. Peptides are involved in supplying nutrition and have sensory functions of taste, solubility, emulsifiability, and various physiological effects [36]. These physiological benefits include anti-cancer activities, enhanced immunity, lowered blood cholesterol and blood pressure levels, and improved calcium homeostasis [14,37]. Enzymatic processes result in soybean protein being partially hydrolyzed into SPs, making it capable of exerting physiological activities [38]. The soy amino acids are well absorbed by the body, and the hydrophobic amino acids are broken down at the C or N terminal ends, which reduces the bitter taste. Moreover, SPs have strong gel-forming properties that, in turn, reinforce its foaming and emulsifying activities [4]. By regulating bile acids reabsorption within digestive systems or organs SPs can effectively lower blood cholesterol levels and decrease low-density lipoprotein (LDL) and fat accumulation, which supports their use for the prevention and treatment of arteriosclerosis [39,40]. Angiotensin-I converting enzyme (ACE) inhibitory peptides in soy proteins lower blood pressure, and antithrombotic peptides, which inhibit platelet aggregation [41]. New functional foods containing SPs that exert physiological effects have been commercialized and marketed in various forms, including health-promoting foods (e.g., functional foods), pharmaceuticals, and cosmetics [22].

Soybean protein is a vital source of energy and contain essential amino acids for living organisms, including animals and especially humans, and has extensively studied [10]. Recently, there has been an increased interest in identifying bioactive peptides from natural sources such as plants. These peptides exhibit unique properties, such as antioxidative, antithrombotic, antimicrobial, immunoregulatory, opiate-like, mineral-binding, hypocholesterolemic, and antihypertensive effects because of their structure, composition, and amino acid sequence [10,42,43,44,45].

### 4.1. Neuroprotective Effects and Improvement of Cognitive Impairment

Hypertension known as high blood pressure is belonged to the dangerous factors causing long-term memory loss progression [46]. However, action mode for hypertension-dependent memory loss is not completely elucidated, and treatment remains insufficient. Plant-based bioactive compounds represent alternative agents to treat human diseases without the side effects often associated with commercial drugs [47]. In spontaneously hypertensive rats (SHR) treated with the SP VHVV (10 mg/kg/oral administration) and ACE inhibitors (oral administered, 5 mg/kg) for 24 weeks, VHVV-treated animals were shown to have upregulated expressions of brain-derived neurotrophic factor (BDNF) related to long-term memory and neuronal survival [48]. These results suggest that VHVV may improve long-term memory and neurogenesis by activating cAMP response element-binding protein (CREB)-mediated downstream proteins as a molecular memory pathway.

SPs have also been reported to have positive effects such as cognitive function improvement, brain wave control, and neurotransmitter control in patients with mild cognitive impairment [49]. In a preclinical study using mice, SPs were shown to have a beneficial effect on age-related cognitive decline. Sixteen-week-old male rats (senescence-accelerated mouse (SAM) prone 8 (SAMP8) and normal aging control SAM resistant 1 (SAMR1)) were divided into four groups (SAMP8 control, SAMP8–SP, SAMR1 control, and SAMR1–SP group), fed for 26 weeks, and evaluated for seven days using a Morris water maze test for cognitive testing. SPs were composed primarily of dipeptides and tripeptides (64.1%), and the rats were treated with 70 g/kg SPs. The results indicated that the escape time from the Morris water maze was significantly reduced in the two groups (SAMP8–SP, SAMR1–SP) that consumed the SPs. Compared with the control group, the SAMP8–SP group showed significantly increased levels of BDNF and neurotrophin-3, which act on neurotrophic factors in the central and peripheral nervous systems of the brain. The expression level of phosphorylated CREB was also upregulated (Table 1) [50]. These results demonstrate that SPs have preventive effects on cognitive impairment through neurotrophic effects and that they play an important role in preventing aging-related cognitive decline and neurodegenerative diseases, such as Alzheimer’s disease.

### 4.2. Modulation of the Cardiovascular System and Blood Pressure

ACE is responsible for converting angiotensin I to vasoconstrictor angiotensin II, as well as the inactivation of the vasodilator bradykinin, which results in the regulation of blood pressure. Therefore, bioactive peptides exhibiting ACE inhibitory effects can be utilized to control blood pressure in individuals with hypertension [21]. Using liquid chromatography-tandem mass spectrometry (LC-MS/MS) of soybean protein hydrolysate digested with thermolysin, five tripeptides (IVF, LLF, LNF, LSW, and LEF) that exhibited ACE inhibitory activity were identified (Table 1) [51].

NWGPLV peptide with ACE inhibitory activity, identified from soy protein hydrolysate, decreased systolic blood pressure in a dose-dependent manner (~100 mg/kg) in an SHR model, leading to an antihypertensive effect [52]. Ovokinin (FRADHPFL) from ovalbumin induced vasorelaxation by attenuating bradykinin B1 receptor and prostacyclin as an endothelium-dependent relaxing factor. Ovokinin (2–7) (RADHPF) exhibited antihypertensive activity by regulating the expression of an unknown receptor and nitric oxide. Both peptides reduced blood pressure in the SHR model when administered orally at a dose of 10 mg/kg [53,54]. IFL and WL from tofu and fermented soybean were also shown to exhibit an ACE inhibitory activity [55]. The peptide HSYNLRQSQVSELKYEGNWGPLVNPESQQGSPRV, produced from soy milk during *Lactobacillus plantarum* C2-mediated fermentation, exerted ACE inhibitory and antioxidant activity [56]. LLPVFK, RLPKPW [57], and PGTAVPK [58] from soybean protein were shown to be associated with antihypertensive effects (Table 1). It is necessary to further develop technologies that retain or enhance the activity of bioactive peptides in soybean foods. Moreover, it is important to understand the pharmacology of these peptides during their passage through the gastrointestinal tract without causing negative effects.

### 4.3. Inhibition of Chronic Kidney Disease Progression

Ingesting fermented soybean and lactic acid together can considerably delay the progression of chronic kidney disease (CKD) [109]. In a meta-analysis of patients with CKD, bean-based products were shown to greatly decrease the level of proteinuria and blood creatinine, inter alia, despite a lower increase in the glomerular filtration rate [110]. As lactic acid affects energy control and anti-inflammatory effects, when used together, it may prevent the release of cytokines, which is one of the main pathological features of CKD that leads to an inflammatory reaction and contributes to kidney damage. Therefore, the appropriate use of lactic acid may serve as an effective strategy for the treatment of kidney disease. Adenine, which is known to induce renal failure, was injected into female rats intraperitoneally to induce CKD. This was followed by the ingestion of fermented soybean together with lactic acid. In the rats with induced CKD, there was an expansion of the renal tubules and interstitial inflammation, as well as a substantial decrease in body weight and food intake. The group that ingested fermented soybean and lactic acid exhibited reduced expression of inflammatory markers, such as interferon gamma (IFN-γ), interleukin 1 (IL-1), IL-6, tumor necrosis factor-alpha (TNF-α), and Toll-like receptor 4 (TLR4). The expression of these markers had previously increased because of adenine administration as well as suppression of renal tubule expansion (Table 1) [111]. Based on these results, it was postulated that the combined ingestion of fermented soybean and lactic acid mitigates the symptoms of renal tubule expansion and interstitial inflammation, thereby suppressing the progression of CKD.

### 4.4. Immunoregulatory Effects

Some immunostimulating peptides have been isolated from enzymatic digests of soybean proteins. These peptides contain specific binding sites on human blood phagocytic cells and stimulate phagocytosis in human and murine macrophages [59]. A tridecapeptide (MITLAIPVNKPGR; also known as soymetide-13) was demonstrated to stimulate phagocytosis of human neutrophils and promote TNF-α expression in mice [59]. An immunostimulating Q (Gln)-abundant peptide was isolated and purified from a soybean-based protein fraction digested with *Rhizopus oryzae*-derived peptidase R. The purified peptide, which was predicted to be located at or near the Q-rich region between residues 202 and 222 of the glycinin G4 subunit, increased the number of CD8(+), CD11b(+), and CD49b(+) cells in C3H/HeN mouse spleen cell cultures. Furthermore, a chemically synthesized Q-rich peptide (QQQQQQKSHGGR) corresponding to residues 202–213 of the glycinin G4 subunit increased the number of CD49b(+), IL-2(+)CD4(+), and interferon-γ(+)CD4(+) cells and stimulated the cytotoxic activity of spleen cells against the K562 human erythroleukemia cell line. These results suggest that the Q-rich domain of the soybean glycinin G4 subunit can stimulate immunoregulatory activity in mouse spleen cells [60]. Soymetide (MITLAIPVNKPGR or LITLAIPVNKPGR), isolated from soymetide [α’ subunit of β-conglycinin (βCG)] digested with trypsin, exhibited immunostimulatory activity as a functional peptide (Table 1) [61]. SPs increased inflammatory stress by reducing white blood cell populations. Moreover, SPs increased immunoglobulin (Ig) M (IgM), IgG, and IgA levels in serum and reduced IL-1β and TNF-α levels, and upon activation, regulated normal T-cell-expressing and -secreting chemokine (C-C motif) ligand 5 in serum. Furthermore, SPs play a beneficial role by enhancing the immune system under conditions of negative nitrogen balance following burn-injury and inhibiting excess inflammation reaction. SPs represent a novel candidate for the treatment and prognosis of patients with burns [112].

### 4.5. Inhibition of Cancer Cell Proliferation by SPs

The ingestion of various anticancer compounds present in soybeans suppresses breast cancer cell proliferation, and certain combinations yield synergistic effects [113]. A total of 12 bioactive factors (seven types reported from the soybean, viz., isoflavone, lunasin, lectin, trypsin inhibitors, saponin, and β-sitosterol) have been assessed for their antiproliferative activities against human breast cancer cells such as MCF-7 and MDA-MB-231. Of these compounds, those that exhibited strong activity were selected for further evaluation regarding whether they had a synergistic effect with bidirectional combination therapy in suppressing human breast cancer cell proliferation. Each compound enhanced the phosphorylation of adenosine monophosphate-activated protein kinase (AMPK) by attenuating the phosphoinositide 3-kinase (PI3K)/protein kinase B (Akt)/mammalian target of rapamycin (mTOR) pathway, resulting in strong inhibition of benign tumor cell proliferation. The activation of AMPK led to suppression of tumor cell invasion and migration through regulation of the cell cycle and inhibition of protein synthesis, leading to anticancer effects. Synergistic effects that substantially increased AMPK phosphorylation were found between genistin and daidzein in MCF-7 cells and between genistein and β-sitosterol as well as between β-sitosterol and genistin in MDA-MB-231 cells. The combinations of these various bioactive factors in soybeans demonstrate a synergistic effect, which inhibits breast cancer cell proliferation [114].

Soybean is a food that is rich in proteins and contains a variety of bioactive peptides. Lunasin (SKWQHQQSCRKQLQGVNLTPCDDDDDDDDDEKHIMEKIQGRGDDDDDDDDDEKHIMEKQ) is a SP that is known to exert various physiological functions, including the prevention and treatment of cancer. High-purity lunasin inhibited the growth of non-small cell lung cancer cells but not that of normal bronchial cells. In a mouse xenotransplantation study, the size of lung tumors was reduced by 63% after 32 days of treatment with 30 mg/kg lunasin. In addition, an immunoblot analysis of the major cell cycle proteins revealed that lunasin can regulate the expression of G1-specific cyclin-dependent kinase (CDK) complex proteins [62]. Furthermore, by inhibiting the phosphorylation of retinoblastoma protein, an important protein associated with the growth of cancer cells, the growth and spread of lung cancer cells was suppressed. These results were the first to show that lunasin can inhibit the spread of non-small cell lung cancer by inhibiting the phosphorylation of retinoblastoma protein [62]. Lunasin also inhibited the proliferation and tumor sphere-forming capacity of HCT-116 colorectal cancer cells by inducing apoptosis and G1 cell cycle-arrest, resulting in increased sub-G0/G1 phase population of tumor cells. Lunasin-induced apoptosis was associated with caspase-3 activation, poly (ADP-ribose) polymerase cleavage, and cell cycle inhibition through the CDK inhibitor p21 (Table 1) [63].

**Figure 1:**
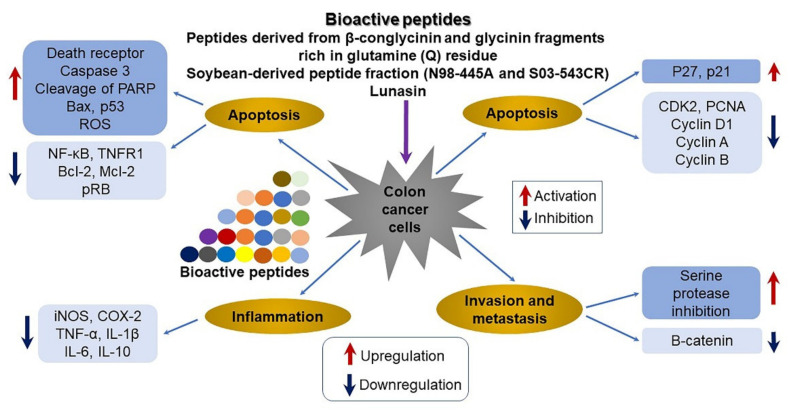
Proposed molecular mechanism associated with bioactive peptides against colorectal cancer. SPs have a critical role in preventive effects by regulating the expression of various genes associated with apoptosis, inflammation, cell cycle arrest, and invasion and metastasis. For apoptosis, these peptides activate death receptor, caspase 3, cleavage of poly (ADP-ribose) polymerase (PARP), Bax, and p53, as well as reactive oxygen species (ROS) accumulation, whereas they inhibit nuclear factor-kappaB (NF-κB), tumor necrosis factor (TNF) receptor type 1 (TNFR1), Bcl-2, Mcl-1, and phosphorylated retinoblastoma protein (pRB). Regarding inflammation, bioactive peptides suppress the expression of genes involved in inducible nitric oxide (NO) synthase (iNOS), cyclooxygenase 2 (COX-2), TNF-α, and IL-1β, IL-6, and IL-10. For cell cycle arrest, they upregulate p27 and p21, whereas they downregulate cyclin-dependent kinase-2 (CDK2), proliferating cell nuclear antigen (PCNA), and cyclins (D1, A, and B). Finally, they cause increased serine protease inhibition and decreased β-catenin associated with invasion and metastasis. This figure is adapted from Avilés-Gaxiola et al. [115].

Enzymatic digestion with pepsin/pancreatin generated 15 SPs. Of the identified peptides, the most potent fraction predominantly contained βCG and glycinin fragments rich in glutamine (Q) residues, including QQQQQGGSQSQ, QEPQESQQ, QQQQQGGSQSQSQKG, and PETMQQQQQQ. Anticancer activity was observed in Caco-2, HT-29, and HCT human colon cancer cells along with anti-inflammatory effects through the suppression of the inflammatory response in lipopolysaccharide (LPS)-induced RAW 264.7 macrophages [67]. The peptide fraction (N98-445A and S03-543CR) derived from soybean protein inhibited the growth of colon cancer (HCT-116) cells by 73%, liver cancer (HepG2) cells by 70%, and lung cancer (NCL-H1299) cells by 68% in a dose-dependent manner (~1000 μg/mL). Thus, SP factions (particularly N98-4445A at 10–50 kDa) may represent an alternative treatment for colon, liver, and lung cancer (Table 1; Figure 1) [115,116].

### 4.6. Anti-Inflammation Properties

Because there are few studies on the autophagy and inflammatory effects of dietary peptides, the LPS-induced inflammation model (RAW 264.7) was used to study the effects of SPs as well as their underlying mechanism of action. The results showed that the SP QRPR decreased the levels of inflammatory cytokines (TNF-α and IL-6) induced by LPS in an inflammatory cell model. QRPR treatment resulted in a time-dependent activation of autophagy by the regulation of the PI3K/Akt/mTOR signaling pathway in LPS-treated RAW 264.7 cells (Table 1) [68]. These results show that QRPR attenuates the inflammatory response of cells by activating autophagy.

Lunasin exerts widespread anti-inflammatory effects by downregulating the expression of nuclear factor kappa B (NF-κB), cytokine, cyclooxygenase 2, and mitogen-activated protein kinase (MAPK) signaling [64,65] in addition to its antioxidant and anticancer effects [65]. The presence of the tripeptide motif, Arg-Gly-Asp (RGD) in lunasin as well as similar peptides contributes to the anti-inflammatory effects by inhibiting αVβ3 intergrin-mediated signaling and downregulating proinflammatory cascades (i.e., Akt-mediated NF-κB pathways via interaction with αVβ3 intergrin) (Table 1) [65,69].

### 4.7. Modulation of Lipid Metabolism

SPs have shown effects on metabolic regulation and physiological properties, such as reduction of cholesterol and triglycerides levels, improvement of lipid metabolism, anti-obesity effects, inhibition of fatty acid (FA) synthase (FAS), and ant-diabetic effects [117]. Several molecules and processes have been the focus of the metabolic effects of bioactive peptides, including intestinal cholesterol micelles, cholesterol metabolism-associated genes that regulate cholesterol, triglyceride metabolism-related genes that reduce triglyceride levels, anti-obesity, dipeptidyl peptidase-IV (DPP-IV), α-amylase, α-glucosidase, and glucose metabolism-related genes that reduce blood glucose levels [117].

**Figure 2:**
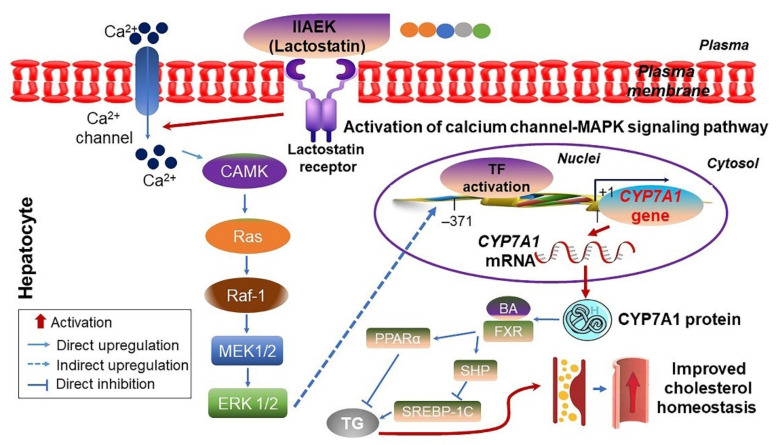
Proposed action mechanism of cholesterol degradation through lactostatin (IIAEK)-mediated *CYP7A1* gene expression in HepG2 cells. Lactostatin improves cellular calcium (Ca^2+^) homeostasis via calcium channel. Subsequently, calcium ions activate calmodulin kinase (CAMK), small GTPase protein (RAS), proto-oncogene serine/threonine-protein kinase (Raf-1), mitogen-activated protein kinase kinase 1 and 2 (MEK1/2), and extracellular signal-regulated protein kinase 1 and 2 (ERK1/2). Upregulated ERK1/2 increases the expression of *CYP7A1* gene associated with cytochrome P450 monooxygenase at the mRNA and protein levels in hepatocytes, which leads to cholesterol homeostasis by regulating the expression of the gene associated with bile acid (BA), famesoid X receptor (FXR)/small heterodimer partner, peroxisome proliferator-activated receptor alpha (PPAR)-α, and sterol regulatory element-binding protein 1C (SREBP-1C). This figure is adapted from Kim et al. [4].

Soybean-derived soystatin (VAWWMY) inhibits the micellar solubility of cholesterol in vitro and cholesterol absorption in vivo, thereby playing an important in bile acid-binding activity [42,70]. In addition, β-lactoglobulin-derived lactostatin (IIAEK) reduces cholesterol levels by modulating the regulatory calcium-channel-related MAPK signaling pathway associated with cholesterol degradation [71]. These activities associated with lactostatin indicate that the extracellular signal-regulated kinase (ERK) pathway and calcium channels are involved in the activity of cholesterol 7α-hydroxylase (CYP7A1), a limiting enzyme for cholesterol degradation and transactivation induced by lactostatin in HepG2 cells (Figure 2) [71]. The regulation of CYP7A1 is important for the prevention and treatment of hypercholesterolemia and atherosclerosis because overexpression of *CYP7A1* was shown to improve hypercholesterolemia and atherosclerosis in animal models [71]. IIAEK constituents such as IAEK, AEK, and EK enhance the expression of *CYP7A1* (Figure 2) [71]. Soy milk β-lactoglobulin-derived β-lactostensin (HIRL) is also capable of reducing serum cholesterol levels through the neurotensin NT_2_ receptor (Table 1) [74].

Lunasin downregulated the expression of genes associated with proprotein convertase subtilisin/kexin type 9 (PCSK9) and upregulated the expression of LDL receptor (LDLR) at the transcriptional and translational levels in HepG2 cells [66]. In ApoE−/− mice, lunasin administration by intraperitoneal injection decreased total cholesterol and LDL-cholesterol levels in the serum by downregulating the expression of PCSK9 and increasing the expression of LDLR and sterol regulatory element-binding protein 2 (SREBP-2) [66]. Soybean glycinin-derived peptides, such as IAVPGEVA, IAVPTGVA, and LPYP, activated the LDLR/SREBP2 pathway in HepG2 cells in vitro, but not in vivo, suggesting that the peptides directly regulate cholesterol metabolism [81]. In contrast, IAEK treatment led to a reduction in cholesterol levels by controlling cholesterol metabolism in vivo through a specific receptor (phosphorylation of ERK), specific proteins [cholesterol 7-alpha-hydroxylase (CYP7A1)] in the cellular membrane, and intracellular Ca^2+^ concentration [72,73]. VVVP, KRES (Apo A-I mimetic peptides), and β-conglycinin-derived peptides (KNPQLR, EIPEKNPQLR, and RKQEEDEDEEQQRE) are new soybean-based bioactive peptides that also attenuate cholesterol and lipid metabolism (Table 1) [42].

Currently, VVVP is the most suitable peptide that attenuates the hypotriglyceridemic mechanism in globin digests. KRES interacts with lipid hydroperoxide (LOOH) to remove LOOH and activates antioxidant enzymes involved in the regulation of high-density lipoprotein (HDL), which attenuates its anti-inflammatory and antiatherogenic effects. βCG-derived peptides inhibit FAS in vitro [42]. In addition, HIRL, DPR, and FVVNATSN exert hypocholesterolemic effects by regulating LDLR expression. However, LPYPR and WGAPSL were shown to increase total plasma cholesterol and LDL cholesterol levels in mice that were fed a low-fat diet [75]. Two hypocholesterolemic peptides (YVVNPDNDEN and YVVNPDNNEN) produced from the LRVPAGTTFYVVNPDNDENLRMIA fragment of soybean βCG enhanced LDL degradation following increased uptake in hepatocytes in vitro. These peptides interacted with the catalytic site of 3-hydroxy-3-methylglutaryl CoA reductase (HMGCoAR) and increased the expression of SREBP2, LDLR, and HMGCoAR in HepG2 cells [76]. GCTLN and QDF isolated from cowpea bean protein [80] and VFVRN derived from chickpea protein [112] exhibited hypolipidemic effects by inhibiting HMGCoAR in vitro and in vivo in a rat model. IAVPGEVA, IAVPTGVA, and LPYP peptides produced by soy glycinin hydrolysis activated the LDLR/SREBP2 pathway and AMPK-ERK1/2 signaling, which enhanced LDL uptake in HepG2 cells in vitro [76]. However, there is limited information regarding the effects of these three peptides on hypocholesterolemia in vivo. Currently, soystatin (VAWWMY) from soybean is the only hypocholesterolemic peptide to show in vivo activity (Table 1).

The peptides VPDPR, APGPR, and VPGPR are rat enterostatins, and APGPR has been found in both humans and mice [77]. VPDPR and APGPR, which are anorectic peptides present in high-fat diets (HFD), showed hypocholesterolemic effects by attenuating cholecystokinin 1 receptor-dependent activity [78,79]. A similar effect was observed for VPDPR and DPR (a fragment of VPDPR) in mice fed an HFD (Table 1) [78]. Although enterostatin has a significant effect on hypocholesterolemia in mice, additional studies are needed to elucidate the effect in humans.

Lupin peptides (LIPKHSDAD, LTFPGSAED, and GDEQSHQDEGVIVR) derived from β-conglutin were shown to exhibit a wide range of biological effects associated with health, including cholesterol regulation, by interacting with and inactivating the HMGCoAR protein in silico [82] and by enhancing LDL uptake following the inhibition of binding between PCSK9 and LDLR in HepG2 cells in vitro [83]. Thus, lupin peptides, particularly LIPKHSDAD and LTFPGSAED, exhibit hypocholesterolemic activity through the LDLR and PCSK9 pathway in HepG2 cells in vitro (Table 1) [84].

VVYP and YPFVV are involved in hypotriglyceridemic action during globulin digestion. In particular, VVVP inhibits fat absorption from the digestive tract by activating hepatic triacylglycerol (TG) lipase and increasing hepatic free FA levels, which leads to rapid attenuation of dietary hypertriglyceridemia [85]. Enterostatin (VPDPR) is associated with hypotriglyceridemic activity by reducing serum triglyceride levels and regulating energy metabolism in rats fed an HFD through the activation of the sympathetic system in brown adipose tissue [87]. Soymorphin-5 (YPFVV), a soy-derived μ-opioid peptide, is also involved in hypotriglyceridemic activity by decreasing triglyceride and glucose levels via activating adiponectin, peroxisome proliferator-activated receptor gamma (PPARγ), and lipid β-oxidation, as well as energy consumption in KKA^Y^ mice. Therefore, this peptide inhibits hyperglycemia without altering plasma insulin levels [88]. Hypotriglyceridemic action is related to FAS, a multicomponent enzyme that catalyzes the synthesis of long-chain FAs through an NADPH-dependent cyclic reaction [117]. The discovery of FAS inhibitors represents an alternative strategy for the prevention of obesity, obesity-related diseases, and cancer. Three peptides derived from βCG (KNPQLR, EIPEKNPQLR, and RKQEEDEDEEQQRE) were shown to inhibit FAS activity in 3T3-L1 adipocytes in vitro [90]. The FAS inhibitory effects of EIPEKNPQLR and RKQEEDEDEEQQRE resulted in the binding of human FAS to the thioesterase domain and by blocking the active site through interactions within the catalytic triangle, hydrophobic groove, and interface cavity of the human FAS thioesterase domain region [90]. Finally, the WE peptide reduced triglyceride and cholesterol accumulation by inducing PPARα translocation through direct interaction with the PPARα ligand-binding domain in lipid-loaded HII4E cells in vitro [86]. Enterostatin (VPDPR) is related to both hypocholesterolemic and hypotriglyceridemic effects, and the peptides APGPR and VPGPR belong to enterostatin (Table 1).

### 4.8. Antiobesity Effects

Obesity is a major cause of health problems in many developed countries. It is associated with a higher incidence of cardiovascular diseases and associated abnormalities. Insulin resistance, hyperinsulinemia, and dyslipidemia contribute to obesity [117]. The dietary intake of soybean βCG reduces serum TG levels and visceral fat in hyperlipidemic humans [118] and exerts anti-obesity effects by promoting postprandial circulating levels of fibroblast growth factor 21 (FGF21) in mice [119]. By analyzing soy protein isolate (SPI) hydrolysates using LC-MS/MS, three lipolysis-stimulating peptides (ILL, LLL, and VHVV) that enhance lipolysis-stimulating effects regardless of gastrointestinal protease activity, which can act as potential anti-obesity agents, were identified (Table 1) [91].

The consumption of soy protein hydrolysate, a peptide mixture containing approximately 80% peptides with a molecular weight of ≤ 500 Da, reduced hepatic TG levels in the Otsuka Long-Evans Tokushima (OLETF) rat model. Moreover, an SP fraction containing KA, VK, and SY inhibited TG synthesis in HepG2 cells, although the in vitro mechanism of action remains unknown. Additionally, the dipeptide SY suppressed ApoB secretion in HepG2 cells (Table 1) [92]. These results suggest that soy protein hydrolysates containing bioactive peptides can reduce serum TG levels.

Soluble soybean protein peptic hydrolysate (SPH) upregulated the expression of insulin-responsive glucose transporter (GLUT)-4 (GLUT4) and insulin-stimulated glucose uptake during adipocyte differentiation in mouse 3T3-L1 cells. SPH enhanced the expression of PPARγ, which is a key regulator of adipocyte differentiation, and increased lipid accumulation in the same cells. Therefore, SPH may have important health benefits based on its effect on obesity-associated metabolic dysfunction [120]. Soybean βCG, a component of SPI, may prevent and attenuate lifestyle-related diseases such as hyperlipidemia and obesity by lowering serum cholesterol and TG levels through limited fat accumulation and inflammatory pathways [118]. Moreover, bioactive peptides (e.g., ILL, LLL, and VHVV) that reduce TG levels have been considered potential therapeutic targets for anti-obesity in 3T3-L1 adipocytes [91]. In particular, VHVV led to the production of lower levels of LDL-cholesterol and TG in mice, which resulted in a reduction in obesity [91]. CKGGRAKDC peptide used for phage display is associated with prohibitin, a multifunctional membrane protein. Targeting of the CKGGRAKDC-GG-KLAKLAKKLAKLAK-mediated proapoptotic peptide to prohibitin resulted in the removal of white fat from the adipose vasculature (Table 1) [93]. To date, no active in vivo anti-obesity compound derived from food proteins has been identified. Thus, identifying an unknown anti-obesity peptide derived from various sources, including soybean, should be a focus for future studies.

### 4.9. Antiarteriosclerosis Effects

The antiatherogenic activity toward blood vessels is another important property of soybean-based bioactive peptides. Apolipoprotein A-I (ApoA-I) is a major component of HDL. Several studies have investigated synthesized ApoA-I mimetic peptides. For example, 4F (DWFKAFYDKVAEKFKEAF) is a mimetic peptide of ApoA-I that was shown to exhibit antiatherogenic, anti-inflammatory, and antioxidative activity and enhance vascular repair in vitro in a rat model of scleroderma [94]. With regard to the antiatherogenic activity, 4F increased HDL formation and cholesterol efflux and decreased lipoprotein oxidation in vitro [94]. It also reduced renal inflammation in LDLR-null mice fed a Western diet; reduced arthritis in a rat model; decreased adiposity, increased adiponectin levels, and improved insulin sensitivity in obese mice; and enhanced HDL inflammatory activities in humans with coronary heart disease (Table 1) [94].

The peptide KRES interacting with lipids can reduce LOOH of lipoproteins during atherosclerosis, increase paraoxonase activity and plasma HDL-cholesterol levels, and exhibit HDL anti-inflammatory properties in ApoE null mice [89]. In addition, the peptide FREL was shown to be associated with HDL levels [89]. Based on these results, peptides capable of binding to lipids can ablate LOOH and elevate the levels of antioxidant enzymes involved in the regulation of HDL, thereby regulating their anti-inflammatory and antiatherogenic effects, regardless of their capacity to produce amphipathic helixes [89]. Dipeptide WH was also associated with antiatherogenic effects by attenuating atherosclerotic lesions (~38% for a WH dose of 100 mg/kg/d) in apo E-deficient mice fed an HFD, which may act as an alternative mechanism to regulate lipid metabolism (Table 1) [95]. The identification of novel hypolipidemic peptides from natural protein materials, including food proteins, may represent an alternative approach to prevent and treat metabolic syndromes with possibly fewer adverse effects [42]. Further studies on animal and human models are required to explore the biological activity and side effects that occur in the management of cholesterol- and lipid-related diseases as well as to understand the underlying molecular mechanisms of action of these functional peptides.

### 4.10. Antidiabetic Effects

Type 2 diabetes (T2D) results from insulin resistance or low insulin production in which the maintenance of glucose homeostasis is dysregulated. There has been considerable interest in natural remedies, such as food constituents, for the management of T2D. DPP-IV, α-amylase, and α-glucosidase are key enzymes that are directly associated with the regulation of blood glucose levels. Bioactive peptides that inhibit the activities of these enzymes may be effective for the control of T2D [96,117]. Because DPP-IV, an enzyme found both in the blood and in the cell membrane, is responsible for the inactivation of incretin hormones, glucagon-like peptide-1 and gastric inhibitory polypeptide, recent studies have focused on food-derived peptides as novel inhibitors [96,117]. DPP-IV inhibitory peptide (IAVPTGVA), identified in soybean-derived protein, may be used for the effective management of T2D and other metabolic diseases (Table 1) [96].

Although the underlying mechanism of action is unclear, βCG, a major source of soy proteins, can regulate blood glucose levels. Adiponectin levels in the plasma and adiponectin receptor 1 mRNA expression in skeletal muscle were higher in βCG-fed Goto-Kakizaki (GK) rats compared with those in casein-fed rats. Phosphorylation of AMPK was activated in βCG-fed GK rats. Subsequently, βCG increased the translocation of GLUT4 to the plasma membrane. Downregulation of sterol regulatory element-binding factor 1 expression, induced by low insulin, increased the expression of hepatic insulin receptor (IR) substrate 2 (IRS2). Thus, consumption of soy βCG improves glucose uptake through AMP kinase activation in skeletal muscle and ameliorates hepatic insulin resistance. These activities may help maintain blood glucose homeostasis in GK rats [121]. Soymorphin-5 (YPFVV) derived from the β-subunit of soybean βCG is a μ-opioid agonist peptide that was shown to exert anxiolytic-like activity by promoting glucose and lipid metabolism in a T2D KKAy mouse model. Soymorphin-5 inhibited hyperglycemia without increasing plasma insulin levels while decreasing plasma and liver TG levels and liver weight. Soymorphin-5 increased plasma adiponectin levels and the expression of genes encoding AdipoR2 (a subtype of the adiponectin receptor involved in stimulating the PPARα pathway and FA β-oxidation), acyl-CoA oxidase, carnitine palmitoyltransferase 1A, and uncoupling protein-2 in the liver (Table 1). These results suggest that soymorphin-5 enhances glucose and lipid metabolism through activation of the adiponectin and PPARα system and increases β-oxidation and energy expenditure [88].

GLUT is involved in the passive uptake of glucose along with its concentration-dependent gradient without energy, and sodium-glucose co-transporter (SGLT) is involved in the uptake of glucose against its concentration gradient with energy [117]. Therefore, glucose transporters, such as GLUT and SGLT are important targets in diabetes treatment. Black bean-derived peptides (AKSPLF, ATNPLF, FEELN, and LSVSVL) were shown to reduce the uptake of glucose in Caco-2 cells and rats [97]. The soybean-derived bioactive peptide aglycin, was effective in controlling hyperglycemia and improving glucose tolerance by restoring insulin signaling transduction through the maintenance of IR and IRS expression at both the transcriptional and translational levels, as well as enhancing the expression of phosphorylated IR (p-IR), p-IRS1, p-Akt, and GLUT4 in C2C12 cells and in streptozotocin/HFD-induced diabetic BALB/c mice (Table 1). These results suggest that oral aglycin administration can attenuate or prevent hyperglycemia by increasing IR signaling in the skeletal muscle of mice [122].

Vglycin, a natural 37-residue polypeptide (VSCNGVCSPFEMPPCGSSACRCIPYGLVVGNCRHPSG) identified in pea seed improved glucose homeostasis and insulin sensitivity by restoring fasting blood glucose levels; impaired insulin signaling; enhanced glucose uptake in hepatocytes through phosphorylation of IR, Akt, total IR, PI3K, glycogen synthase kinase 3, and GLUT4 in Wistar rats fed an HFD; and protected pancreatic cells from streptozotocin-induced apoptosis. It also reduced food intake and body weight. In contrast, it did not affect insulin synthesis and release in impaired β-cells (Table 1). These results suggest that vglycin could be an alternative for the prevention and treatment of T2D [98].

### 4.11. Skin Protection Effect of Soybean Oligopeptides against Ultraviolet Radiation

A research team conducted clinical and cellular studies [123] on the protective effect and mechanism of action of soybean oligopeptides against acute light damage to the skin induced by ultraviolet B (UVB) radiation. In a clinical trial, the skin erythema index in the UVB-irradiated group was significantly increased compared with that in the negative control group; however, the erythema index was significantly decreased in the group treated with soybean oligopeptides after UVB irradiation compared with that in the UVB group. In addition, on the 1st and 3rd days following UVB irradiation, skin stratum corneum hydration was significantly reduced compared with that in the negative control, but it was confirmed that soybean oligopeptides inhibited moisture loss due to UVB radiation. In cellular experiments, UVB radiation increased sunburn and the number of apoptotic cells, but this was significantly decreased in the group treated with soybean oligopeptide. UVB radiation increased the expression of p53 and Bax proteins and decreased the expression of Bcl-2 protein. In contrast, soy oligopeptides reduced the expression of p53 and Bax and increased the expression of Bcl-2 protein to prevent UVB radiation-induced light damage. Based on these results, soybean oligopeptides may protect human skin from damage caused by UV radiation [123].

### 4.12. Antioxidative Properties

The effect of SPs has rarely been studied in patients with hypertension. Black SPs have been shown to possess antioxidative properties. Following eight weeks of black SP dietary supplementation (4.5 g/d) in patients with hypertension, black SP improved redox homeostasis, including a decrease in plasma malondialdehyde (MDA) and urinary 8-epi-prostaglandin F_2__α_ levels and an increase in plasma superoxide dismutase (SOD) activity. Furthermore, systolic blood pressure changes were negatively correlated with the change in serum total nitric oxide (NO) level. Elevated serum NO level was negatively correlated with serum ACE activity and MDA level was positively correlated with enhanced SOD activity [124]. The ACE-inhibitory peptide HHL, derived from a Korean fermented soybean product, exhibited antihypertensive activity in vivo [99]. Similarly, black SP contains HHL and higher levels of arginine (9.63%), a metabolic precursor of the potent vasodilator NO (Table 1) [125]. Thus, black SP exhibits blood pressure-lowering effects by improving oxidative stress-induced redox status, enhancing NO production, and reducing ACE activity.

Treatment of *Caenorhabditis elegans* under oxidative stress with the novel antioxidant peptide FDPAL (10 mM) induced a longevity effect by attenuating its direct reactive oxygen species (ROS)-scavenging activity and indirect free radical-scavenging activity (FRSA) through high expression of tolerance-associated genes, such as *SOD3* [100]. Alkaline phosphatase hydrolysates of soy protein, containing the peptides LLPLPVLK, SWLRL, and WLRL, exhibited antioxidant activity and inhibited α-glucosidase, DPP-IV, and ACE activity [101]. Soybean hydrolysates with a molecular weight of < 3 kDa prepared by chymotrypsin digestion exhibited reduced liposome oxidation and also showed the highest free FRSA [126]. In addition to molecular weight, the antioxidant activity and FRSA of soy hydrolysates corresponded with their amino acid composition, in particular, increased content of Phe and decreased content of Lys (Table 1) [127].

In general, thiol-containing peptides (TCPs) that contain Tyr (Y), Met (M), His (H), Lys (K), Cys (C), and Trp (W) are effective antioxidants and include glutathione (GSH), phytochelatins and metallothionein in the organism [104]. In a recent study, novel TCPs exhibited antioxidant activity. A high content of Cys in TCPs contributed to the antioxidant activities following direct binding with radicals. The IC_50_ values of TCPs for 2,2–diphenyl–1–picrylhydrazyl (DPPH), hydroxyl radical, superoxide anion radical, and reducing power were 0.1 ± 0.004 mg/mL, 1.49 ± 0.07 mg/mL, 0.084 ± 0.004 mg/mL, and 0.38 ± 0.01 mg/mL, respectively. Meanwhile, the IC_50_ values of GSH for these parameters were 0.03 ± 0.001 mg/mL, 0.36 ± 0.014 mg/mL, 0.052 ± 0.003 mg/mL, and 0.57 ± 0.031 mg/mL, respectively. Except for reducing power, the TCP values were higher than those of GSH (Table 1) [104]. The DPPH value of TCPs was higher than that of GSH (0.03 mg/mL) but lower than that of enzymatic rapeseed protein (0.45–0.6 mg/mL) [124], chickpea protein hydrolysate (0.57–1.2 mg/mL) [128], and mung bean (28.13–35.68 μM/g) [129]. In addition, the scavenging of hydroxyl and superoxide radicals of the TCPs was lower than that of GSH [104] but comparatively higher than that of chicken skin protein hydrolysates (0.57–1.22 mg/mL and 0.7–1.3 mg/mL, respectively) [130] and egg white protein hydrolysates (0.2–0.57 mg/mL and 0.11–0.36 mg/mL, respectively) [131]. Thus, TCPs show powerful antioxidant activity against hydroxyl and superoxide radicals by neutralizing oxidative stress-induced ROS generation in aerobic organisms.

Oxidative stress is associated with various diseases, including T2D, neurodegenerative diseases, immunosuppression, cancer, aging, obesity, cardiometabolic dysfunctions, and metabolic syndromes [132]. Lunasin exhibits antioxidant activity by detoxifying ROS generated in LPS-induced RAW 267.7 macrophages in a dose-dependent manner, which could prevent the ROS-mediated oxidation of cellular components [132]. Peptides (GNPDIEHPE, TNDRPSIG, SVIKPPTDE, VIKPPTDE, GNPDIEHPET, LVPPQESQ, EITPEKNPQ, TLVNNDDRDS, NSQHPEL, and FEEPQQPQ) derived from full-fat soybean flakes enhanced DPPH, 2,2′-azino-bis(3-ethylbenzothiazoline-6-sulfonic acid (ABTS), reducing power, iron chelation, and inhibition of ROS production in Caco-2 cells (Table 1) [105]. Moreover, unknown bioactive peptides derived from a soy protein fraction and soy industrial effluents [133], black soy source [134], raw soybeans [135,136], SPI [137], black soybean milk [138], and soy protein [139] exhibited significant antioxidant activity by enhancing DPPH, hydroxyl radical-scavenging activity, ABST, ferric reducing ability of plasma, oxygen radical antioxidant capacity, thiobarbituric acid reactive substance, reducing power, nitroblue tetrazolium inhibition, lipid oxidation inhibition, and metal ion (Cu^2+^ and Fe^2+^) chelation. Additionally, peptides produced by *Lactobacillus* spp.-mediated soy milk fermentation exhibited antioxidant activity by enhancing the abovementioned activity-related factors [140,141,142,143,144]. Peptides produced by fermentation based on *Lactobacillus* spp.-mediated soybean meal extract [145] and *Chryseobacterium* sp.-mediated SPI [146] exhibited significant antioxidant activity.

Short (LH) and medium (VNPESQQGSPR) peptides derived from soybean protein play an important role in antioxidant activity in vitro. Cells pretreated with both peptides reduced ROS levels compared with cells treated with hydrogen peroxide (H_2_O_2_). Compared with the medium peptide, the short peptide exhibited a higher protective effect against oxidative stress generated by H_2_O_2_, almost restoring to the levels of control cells (without peptide pretreatment) (Table 1). These results suggest that soybean-derived bioactive peptides exhibit significant antioxidant activities, particularly short peptides [102]. Selenium (Se)-containing peptides [SFQ(K)SeM and SeCPEE] from soybean rich in Se also exhibited antioxidant properties by upregulating liver SOD and GSH peroxidase-1 and downregulating aspartate aminotransferase, alanine aminotransferase, and NF-κB levels through the regulation of MAPK and NF-κB signaling pathways in the presence of Se-containing peptides, which enhanced the protective effects against diseases associated with oxidative stress (Table 1) [103]. These studies show that most antioxidative properties are associated with known/unknown SPH or SPH products. With growing interest in functional food science technology, soy-based products and byproducts have received attention as a safer alternative to preventive and therapeutic applications. Soy products including SPs has been associated with the preventing or reducing the risk of various diseases, such as memory loss, hypertension, obesity, diabetes, inflammation, cancer, skin damage, and cardiovascular and kidney diseases, through the beneficial health effects exerted through its interactions with cell receptors, interference with the cell cycle, regulation of enzymatic activity, or role as a hormone, which can lead to the cellular homeostasis involved in metabolic process or signal induction [126].

### 4.13. Soybean Proteins and Control of Increased Gut Microbial Activity Caused by Probiotics

Based on the type of dietary protein, it is possible to control the effects of probiotics on gut fermentation. Although the use of common and widely known probiotics, such as dietary fiber and olive oil by gut microorganisms, results in the influx of 4–10 g of undigested protein, it was determined that mixing certain proteins can impact the fermentation process. Soybean protein, meat (beef + pork) protein, and fish protein were mixed with probiotics (cellulose or raffinose) and fed to rats for four weeks, and the resulting metabolic processes were analyzed. Rats that consumed raffinose as the probiotic instead of cellulose had an increased number of *Lactobacillus* spp., regardless of the type of protein. Compared with rats that ingested other animal-based protein combinations, rats that ingested soybean protein had an increased abundance of *Bifidobacterium* spp., comparable to the amount in the culture medium. This suggests that soybean protein supplied large amounts of nutrients for bacterial growth. Additionally, the soybean protein combination resulted in a significant increase in appendix IgA concentration compared with other combinations. This may be interpreted positively as a way to prevent the invasion of pathogenic bacteria through the colon [147]. Based on these results, it was confirmed that the effect of probiotics on gut fermentation and microbiota is controlled through dietary protein content, and there is a difference between plant-based and animal-based proteins in these effects [147].

### 4.14. Antimicrobial and Antiviral Effects

Antimicrobial resistance is a significant concern in both the medical and food industry worldwide because microorganisms cause various diseases (e.g., inflammation-mediated diseases and food poisoning) [106]. The synthesized SPs PGTAVFK and IKAFKEATKVDKVVVLWTA have shown antimicrobial effects against *Pseudomonas aeruginosa* and *Listeria monocytogenes* above a dose of 625 μΜ and 37.2 μΜ, respectively [106]. The peptides KHPHGRSYKTKLRILA, LRFRAPAPVLRRIAKR, and HTSKALLDMLKRLGK, produced from *Bacillus subtilis* E20-fermented soybean meal, exhibit antimicrobial activity against *Vibrio alginolyticus* and *V*. *parahaemolyticus*, which could be a biofunctional source to prevent vibriosis in shrimp aquaculture (Table 1) [107].

A P13 (ALPEEVIQHTFNLKSQ) peptide produced by *B*. *licheniformis* KN1G-mediated soybean fermentation contains the human ACE2 cell receptor-binding domain. The P13-human TLR4/myeloid differentiation factor (MDF2) complex is known for its critical role in the cytokine storm based on in silico analysis. These results suggest that ALPEEVIQHTFNLKSQ has greater potential to be applied as an antiviral agent and used as a prophylaxis agent against viral diseases, including coronavirus disease 2019 and severe acute respiratory syndrome coronavirus 2 (SARS-CoV-2) infection (Table 1) [108]. As the production of various antiviral peptides is dependent on the culture conditions of species as well as the strain, further studies of selected peptides using molecular docking systems are needed to demonstrate that the peptide interacts with the active residues of SARS-CoV-2 spike glycoprotein (S1) receptor-binding domain and the human TLR4/MDF2 complex. The abovedescribed functionality of soybean-derived bioactive peptides is summarized in Table 1.

## 5. Economic Feasibility and Application of Soybean-Derived Bioactive Peptides

Milk proteins (milk, whey, and sodium caseinate) or peptides derived from milk proteins are highly sensitive to changes in supply and have relatively high price fluctuations. Historically, the volatility of the milk protein market has made predicting milk price and supply difficult. Food manufacturing companies with overly high reliance on milk protein often suffer financially from repeated cost fluctuations [148]. In contrast, soy protein isolate or soy-derived bioactive peptides provide the financial benefits of relatively stable supply and cost, which can effectively supplement the volatility of milk protein products. The prediction of cost and supply for SPs is much easier than that for milk protein products, which makes cost management easy and increases the profitability of products. High-quality SPs may be used as financial substitutes for casein, milk protein concentrate, skim milk powder, whole milk powder, and whey protein-derived peptide material [149,150].

The effects of SPs can easily be sensed, as these are substances with rapid absorption. Thus, research on SPs is directed toward ensuring consumer experience. Research on commercial products is focused on products that have SP content of 4000 mg or more, at which they can be felt by consumers. This is because customer experience cannot be proven without evidence of the effect felt from products that are accessible. Recently, the development of soy products for fatigue recovery in businessmen has been gaining attention. These studies intend to demonstrate the effect of SPs in businessmen, who are most vulnerable to psychological and physical fatigue, through in-person experience [10,151,152]. In addition, a new marketing strategy using the concept of household essential drugs, which are always available in households and accessible at any time, is being attempted. Furthermore, there has been a recent shift in the focus of consumers and markets toward high functional SPs upon their start of sale [153,154]. SPs currently in market have diverse applications such as in sports drinks, meal replacements, diet meals, and health supplements. SP material market has recently been experiencing rapid global growth as consumers have become increasingly aware of the benefits of soy peptides, including its nutritional and physiological effects, effects on physical fatigue related to absorption rate and sports nutrition, and effect on psychological fatigue related to stress [10,151,155]. In particular, the main attraction of products containing SPs currently available in market is fatigue recovery. In contrast to amino acid products that are mostly marketed through concepts of muscle fatigue relief and increased muscle strength for sportsmen, SP products distinguish themselves through the concept of fatigue recovery [156].

## 6. Conclusions and Perspectives

Traditional fermented foods prepared from soybeans or various peptides derived from soybean hydrolysates may be used as functional food materials to enhance antitumor (e.g., lunasin), anti-inflammatory, cognitive function, skin protection, and antioxidant activities. Because studies on the bioregulatory functions of SPs are at a nascent stage, structural determination of the peptides, identification of the mechanism of action, and large-scale expression by microorganisms should be conducted. This will lead to the discovery of new SPs with various physiological activities that may be utilized in the development of various bioregulatory functional foods, contribute to the improvement of public health, and further development of the food and pharmaceutical-related industries. These findings of this study suggest that SPs are an important resource that have potential applications in the nutraceutical, bioactive material, and clinical medicine fields, as well as for cosmetic and health care products.

SPs are known to exert many physiologically activities that remain to be elucidated. The results of various experiments at the cell and animal levels have not yet been fully linked to the effects and efficacy of human experiments and have not been fully interpreted. To overcome these limitations, it is necessary to discover bioactive peptides that maintain biological safety and pH stability in the gastrointestinal tract using human-based experiments. Furthermore, studies on the mechanism of action of these peptides should be conducted in parallel.

Until recently, it was thought that soybean protein was broken down into peptides and then converted into amino acids and absorbed by the body. SPs, which have the potential to be absorbed into the body and exhibit various functions, have attracted research interest because of their potential applications in human health. In the future, additional physiologically activities will be revealed, and accordingly, the benefits of SPs to human health will be further expanded.

## Figures and Tables

**Table 1:** Representative beneficial effects of soybean-derived bioactive peptides in humans.

Peptide Sequence	Biological Effect	Reference
VHVV	Neuroprotection	[48]
IVF, LLF, LNF, LSW, LEF	ACE inhibitory	[51]
NWGPLV	ACE inhibitory	[52]
FRADHPFL, RADHPF	Lowering blood pressure and vasorelaxation	[53,54]
IFL, WL	ACE inhibitory	[55]
HSYNLRQSQVSELKYEGNWGPLV NPESQQGSPRV	ACE inhibitory and antioxidant	[56]
FRADHPFL, RADHPF	Lowering blood pressure and vasorelaxation	[53,54]
LLPVFK, RLPKPW	Anti-hypertensive	[57]
PGTAVPK	Anti-hypertensive	[58]
MITLAIPVNKPGR	Immunoregulatory	[59]
QQQQQQKSHGGR	Immunoregulatory	[60]
LITLAIPVNKPGR	Immunoregulatory	[61]
Lunasin (KWQHQQSCRKQLQGVNLTPCDD DDDDDDDEKHIMEKIQGRGDDDD DDD DDEKH IMEKIQ)	Anticancer, anti-inflammatory, antioxidant and cholesterol regulation	[62,63,64,65,66]
QQQQQGGSQSQ, QEPQESQQ, QQQQQGGSQSQSQKG, PETMQQQQQQ,	Anti-cancer	[67]
QRPR	Anti-inflammatory	[68]
RGD	Anti-inflammatory	[65,69]
Soystatin (VAWWMY)	Cholesterol regulation	[42,70]
Lactostatin (IIAEK)	Cholesterol regulation	[71,72,73]
Lactostensin (HIRL)	Cholesterol regulation	[74]
LPYPR, WGAPSL	Cholesterol regulation	[75]
IAVPGEVA, IAVPTGVA, LPYP	Cholesterol regulation and lipid metabolism	[76]
HIRL, DPR, FVVNATSN	Hypocholesterolemic	[75]
YVVNPDNDEN, YVVNPDNNEN	Hypocholesterolemic	[76]
DPR, VPDPR, APGPR, VPGPR	Hypocholesterolemic	[77,78,79]
GCTLN, QDF	Hypolipidemic	[80]
IAVPGEVA, IAVPTGVA, LPYP	Hypolipidemic	[81]
Lupin peptides (LIPKHSDAD, LTFPGSAED, GDEQSHQDEGVIVR)	Hypolipidemic and hypocholesterolemic	[82,83,84]
VVVP, YPFVV	Hypotriglyceridemic	[42,85]
WE	Hypocholesterolemic and hypotriglyceridemic	[86]
Enterostatin (VPDPR)	Hypocholesterolemic and hypotriglyceridemic	[87]
Soymorphin-5 (YPFVV)	Hypotriglyceridemic and anti-diabetic	[88]
KRES, FREL	Hypotriglyceridemic antioxidant, anti-inflammatory and anti-atherogenic	[42,89]
KNPQLR, EIPEKNPQLR, RKQEEDEDEEQQRE	FAS inhibitor	[90]
KNPQLR, EIPEKNPQLR, RKQEEDEDEEQQRE	Hypotriglyceridemic and FAS inhibitor	[42]
ILL, LLL, VHVV	Lipolysis and anti-obesity	[91]
SY	Anti-obesity	[92]
CKGGRAKDC	Anti-obesity	[93]
DWFKAFYDKVAEKFKEAF	Anti-arteriosclerosis	[94]
FREL	Anti-arteriosclerosis	[89]
WH	Anti-arteriosclerosis	[95]
IAVPTGVA	Anti-diabetic	[96]
AKSPLF, ATNPLF, FEELN, LSVSVL	Anti-diabetic	[97]
Vglycin (VSCNGVCSPFEMPPCGSS ACRCIPYGLVVGNCRHPSG)	Anti-diabetic	[98]
HHL	ACE inhibitory and antioxidant	[99]
FDPAL	Antioxidant	[100]
LLPLPVLK, SWLRL, WLRL	Antioxidant	[101]
LH, VNPESQQGSPR	Antioxidant	[102]
Selenium (Se)-containing peptide [SFQ(K)SeM and SeCPEE]	Antioxidant	[103]
Thiol-containing peptides containing Tyr, Met, His, Lys, and Cys	Antioxidant	[104]
GNPDIEHPE, TNDRPSIG, SVIKPPTDE, VIKPPTDE, GNPDIEHPET, LVPPQESQ, EITPEKNPQ, TLVNNDDRDS, NSQHPEL, FEEPQQPQ	Antioxidant	[105]
PGTAVFK, IKAFKEATKVDKVVVLWTA	Antimicrobial	[106]
KHPHGRSYKTKLRILA, LRFRAPAPVLRRIAKR, HTSKALLDMLKRLGK	Antimicrobial	[107]
ALPEEVIQHTFNLKSQ	Antiviral	[108]
